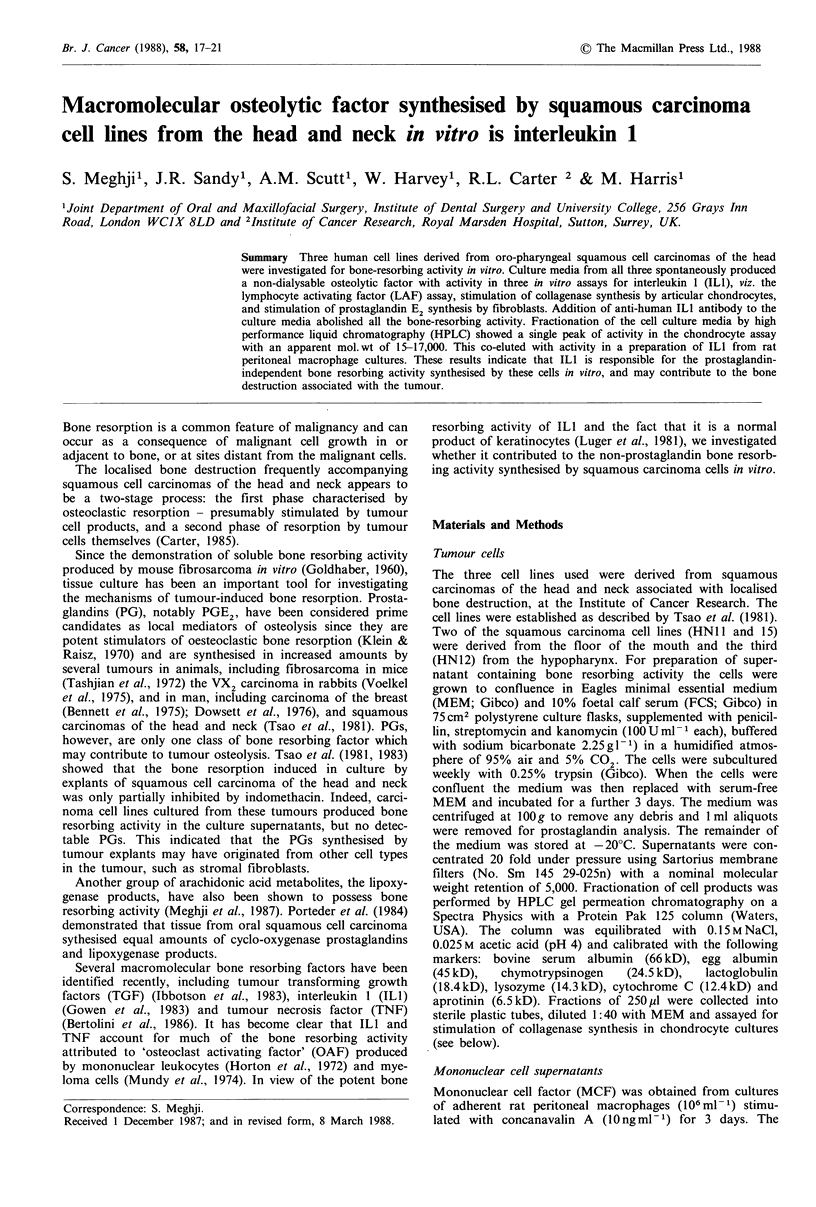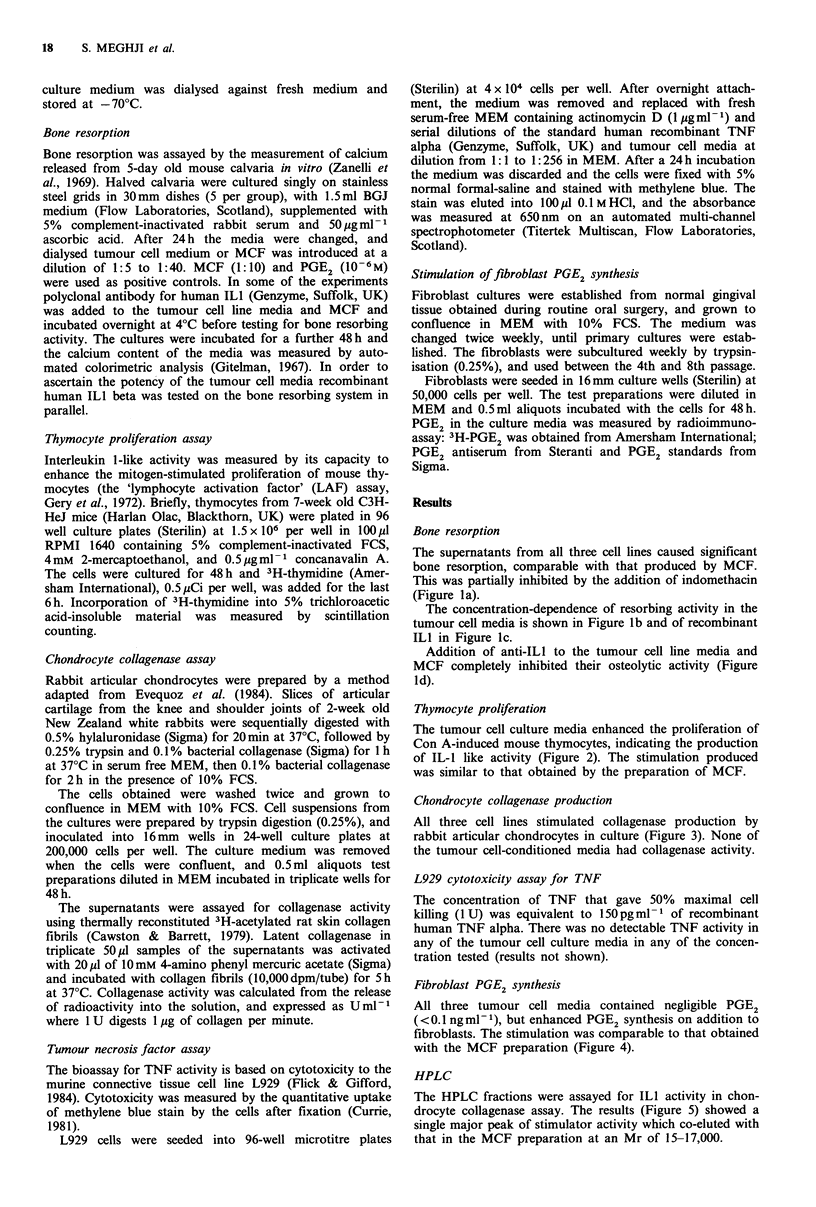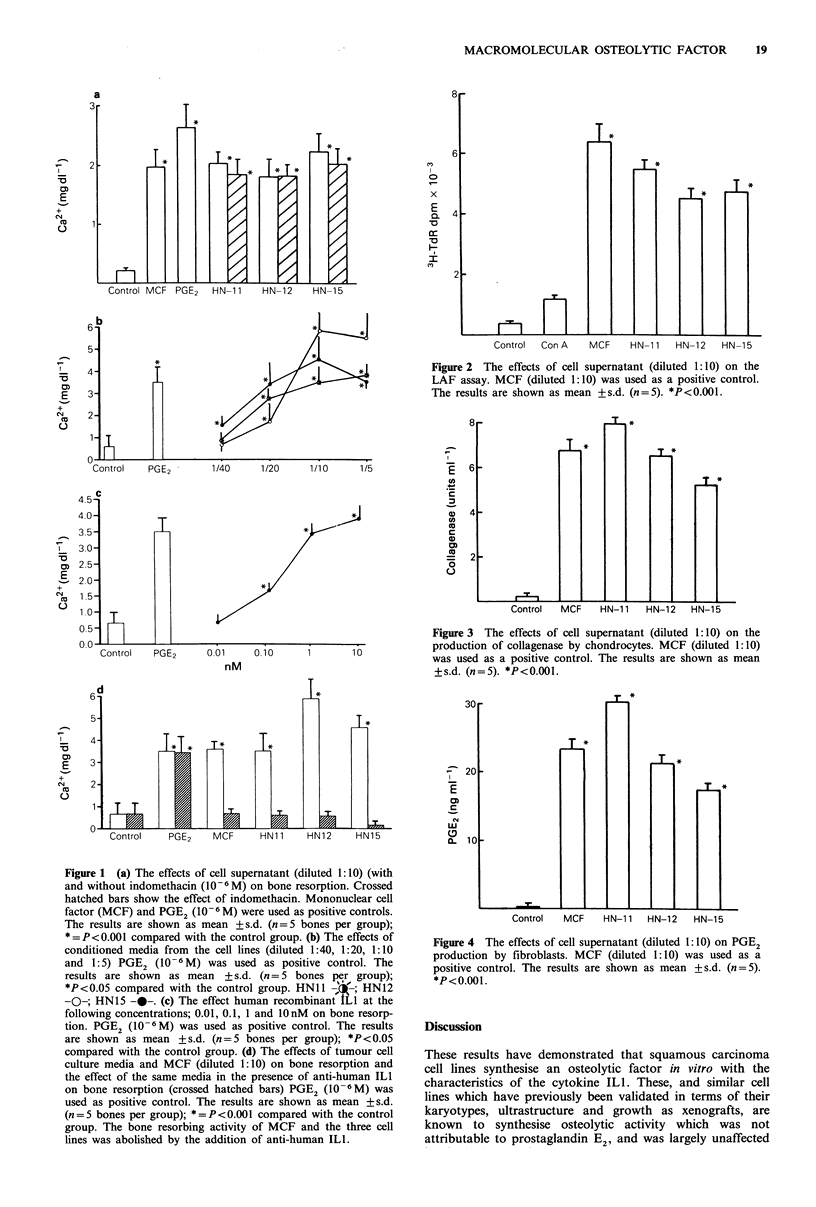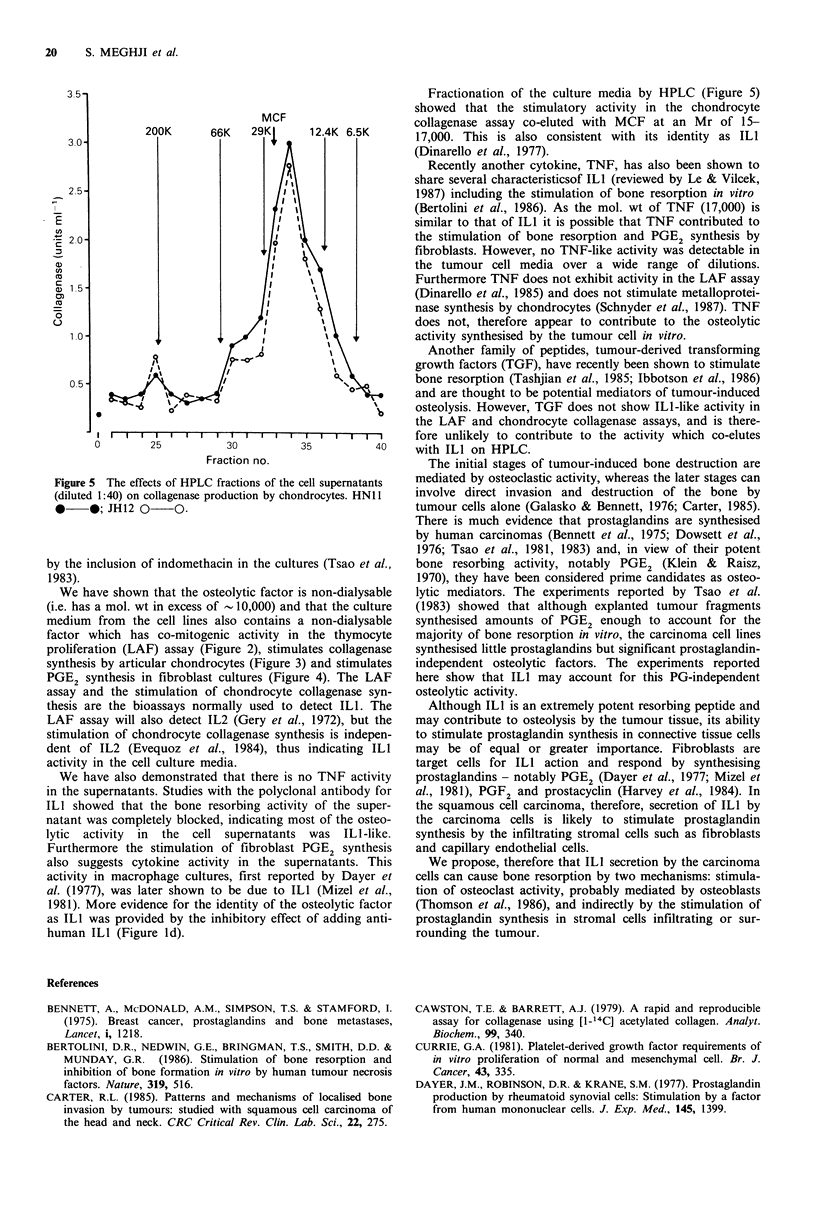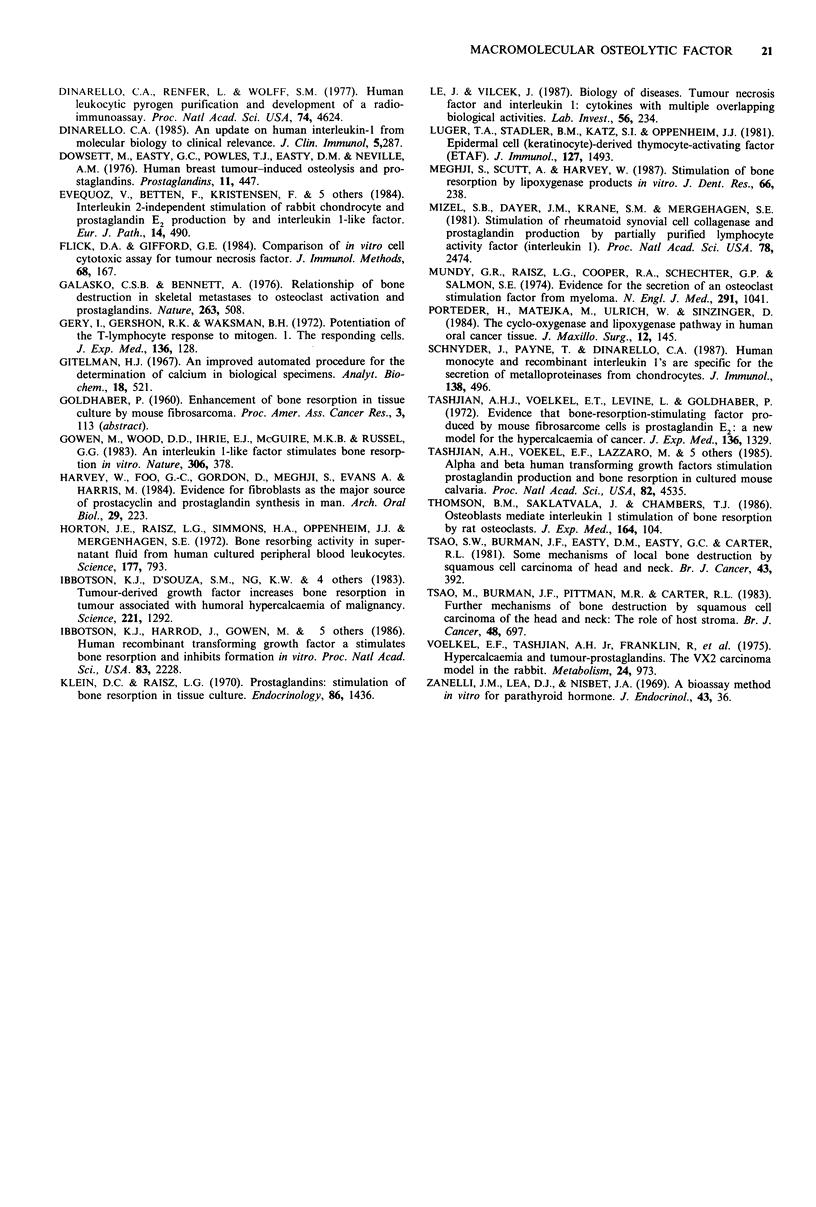# Macromolecular osteolytic factor synthesised by squamous carcinoma cell lines from the head and neck in vitro is interleukin 1.

**DOI:** 10.1038/bjc.1988.153

**Published:** 1988-07

**Authors:** S. Meghji, J. R. Sandy, A. M. Scutt, W. Harvey, R. L. Carter, M. Harris

**Affiliations:** Joint Department of Oral and Maxillofacial Surgery, Institute of Dental Surgery and University College, London, UK.

## Abstract

Three human cell lines derived from oro-pharyngeal squamous cell carcinomas of the head were investigated for bone-resorbing activity in vitro. Culture media from all three spontaneously produced a non-dialysable osteolytic factor with activity in three in vitro assays for interleukin 1 (IL1), viz. the lymphocyte activating factor (LAF) assay, stimulation of collagenase synthesis by articular chondrocytes, and stimulation of prostaglandin E2 synthesis by fibroblasts. Addition of anti-human IL1 antibody to the culture media abolished all the bone-resorbing activity. Fractionation of the cell culture media by high performance liquid chromatography (HPLC) showed a single peak of activity in the chondrocyte assay with an apparent mol.wt of 15-17,000. This co-eluted with activity in a preparation of IL1 from rat peritoneal macrophage cultures. These results indicate that IL1 is responsible for the prostaglandin-independent bone resorbing activity synthesised by these cells in vitro, and may contribute to the bone destruction associated with the tumour.


*Br. J. Cancer* (1988), **58**, 17–21 © The Macmillan Press Ltd., 1988

### Macromolecular osteolytic factor synthesised by squamous carcinoma cell lines from the head and neck *in vitro* is interleukin 1

S. Meghji[1], J.R. Sandy[1], A.M. Scutt[1], W. Harvey[1], R.L. Carter [2] & M. Harris[1]

[1]*Joint Department of Oral and Maxillofacial Surgery, Institute of Dental Surgery and University College, 256 Grays Inn Road, London WC1X 8LD and* [2]*Institute of Cancer Research, Royal Marsden Hospital, Sutton, Surrey, UK.*

**Summary** Three human cell lines derived from oro-pharyngeal squamous cell carcinomas of the head were investigated for bone-resorbing activity *in vitro*. Culture media from all three spontaneously produced a non-dialysable osteolytic factor with activity in three *in vitro* assays for interleukin 1 (IL1), *viz.* the lymphocyte activating factor (LAF) assay, stimulation of collagenase synthesis by articular chondrocytes, and stimulation of prostaglandin $E_2$ synthesis by fibroblasts. Addition of anti-human IL1 antibody to the culture media abolished all the bone-resorbing activity. Fractionation of the cell culture media by high performance liquid chromatography (HPLC) showed a single peak of activity in the chondrocyte assay with an apparent mol. wt of 15–17,000. This co-eluted with activity in a preparation of IL1 from rat peritoneal macrophage cultures. These results indicate that IL1 is responsible for the prostaglandin-independent bone resorbing activity synthesised by these cells *in vitro*, and may contribute to the bone destruction associated with the tumour.

Bone resorption is a common feature of malignancy and can occur as a consequence of malignant cell growth in or adjacent to bone, or at sites distant from the malignant cells.

The localised bone destruction frequently accompanying squamous cell carcinomas of the head and neck appears to be a two-stage process: the first phase characterised by osteoclastic resorption – presumably stimulated by tumour cell products, and a second phase of resorption by tumour cells themselves (Carter, 1985).

Since the demonstration of soluble bone resorbing activity produced by mouse fibrosarcoma *in vitro* (Goldhaber, 1960), tissue culture has been an important tool for investigating the mechanisms of tumour-induced bone resorption. Prostaglandins (PG), notably $PGE_2$, have been considered prime candidates as local mediators of osteolysis since they are potent stimulators of oesteoclastic bone resorption (Klein & Raisz, 1970) and are synthesised in increased amounts by several tumours in animals, including fibrosarcoma in mice (Tashjian *et al.*, 1972) the $VX_2$ carcinoma in rabbits (Voelkel *et al.*, 1975), and in man, including carcinoma of the breast (Bennett *et al.*, 1975); Dowsett *et al.*, 1976), and squamous carcinomas of the head and neck (Tsao *et al.*, 1981). PGs, however, are only one class of bone resorbing factor which may contribute to tumour osteolysis. Tsao *et al.* (1981, 1983) showed that the bone resorption induced in culture by explants of squamous cell carcinoma of the head and neck was only partially inhibited by indomethacin. Indeed, carcinoma cell lines cultured from these tumours produced bone resorbing activity in the culture supernatants, but no detectable PGs. This indicated that the PGs synthesised by tumour explants may have originated from other cell types in the tumour, such as stromal fibroblasts.

Another group of arachidonic acid metabolites, the lipoxygenase products, have also been shown to possess bone resorbing activity (Meghji *et al.*, 1987). Porteder *et al.* (1984) demonstrated that tissue from oral squamous cell carcinoma sythesised equal amounts of cyclo-oxygenase prostaglandins and lipoxygenase products.

Several macromolecular bone resorbing factors have been identified recently, including tumour transforming growth factors (TGF) (Ibbotson *et al.*, 1983), interleukin 1 (IL1) (Gowen *et al.*, 1983) and tumour necrosis factor (TNF) (Bertolini *et al.*, 1986). It has become clear that IL1 and TNF account for much of the bone resorbing activity attributed to 'osteoclast activating factor' (OAF) produced by mononuclear leukocytes (Horton *et al.*, 1972) and myeloma cells (Mundy *et al.*, 1974). In view of the potent bone resorbing activity of IL1 and the fact that it is a normal product of keratinocytes (Luger *et al.*, 1981), we investigated whether it contributed to the non-prostaglandin bone resorbing activity synthesised by squamous carcinoma cells *in vitro*.

Correspondence: S. Meghji.
Received 1 December 1987; and in revised form, 8 March 1988.

### Materials and Methods

### *Tumour cells*

The three cell lines used were derived from squamous carcinomas of the head and neck associated with localised bone destruction, at the Institute of Cancer Research. The cell lines were established as described by Tsao *et al.* (1981). Two of the squamous carcinoma cell lines (HN11 and 15) were derived from the floor of the mouth and the third (HN12) from the hypopharynx. For preparation of supernatant containing bone resorbing activity the cells were grown to confluence in Eagles minimal essential medium (MEM; Gibco) and 10% foetal calf serum (FCS; Gibco) in $75\,cm^2$ polystyrene culture flasks, supplemented with penicillin, streptomycin and kanomycin ($100\,U\,ml^{-1}$ each), buffered with sodium bicarbonate $2.25\,g\,l^{-1}$) in a humidified atmosphere of 95% air and 5% $CO_2$. The cells were subcultured weekly with 0.25% trypsin (Gibco). When the cells were confluent the medium was then replaced with serum-free MEM and incubated for a further 3 days. The medium was centrifuged at 100*g* to remove any debris and 1 ml aliquots were removed for prostaglandin analysis. The remainder of the medium was stored at $-20°C$. Supernatants were concentrated 20 fold under pressure using Sartorius membrane filters (No. Sm 145 29-025n) with a nominal molecular weight retention of 5,000. Fractionation of cell products was performed by HPLC gel permeation chromatography on a Spectra Physics with a Protein Pak 125 column (Waters, USA). The column was equilibrated with 0.15 M NaCl, 0.025 M acetic acid (pH 4) and calibrated with the following markers: bovine serum albumin (66 kD), egg albumin (45 kD), chymotrypsinogen (24.5 kD), lactoglobulin (18.4 kD), lysozyme (14.3 kD), cytochrome C (12.4 kD) and aprotinin (6.5 kD). Fractions of 250 $\mu$l were collected into sterile plastic tubes, diluted 1:40 with MEM and assayed for stimulation of collagenase synthesis in chondrocyte cultures (see below).

### *Mononuclear cell supernatants*

Mononuclear cell factor (MCF) was obtained from cultures of adherent rat peritoneal macrophages ($10^6\,ml^{-1}$) stimulated with concanavalin A ($10\,ng\,ml^{-1}$) for 3 days. The culture medium was dialysed against fresh medium and stored at −70°C.

*Bone resorption*

Bone resorption was assayed by the measurement of calcium released from 5-day old mouse calvaria *in vitro* (Zanelli *et al.*, 1969). Halved calvaria were cultured singly on stainless steel grids in 30 mm dishes (5 per group), with 1.5 ml BGJ medium (Flow Laboratories, Scotland), supplemented with 5% complement-inactivated rabbit serum and $50\,\mu g\,ml^{-1}$ ascorbic acid. After 24 h the media were changed, and dialysed tumour cell medium or MCF was introduced at a dilution of 1:5 to 1:40. MCF (1:10) and $PGE_2$ ($10^{-6}$M) were used as positive controls. In some of the experiments polyclonal antibody for human IL1 (Genzyme, Suffolk, UK) was added to the tumour cell line media and MCF and incubated overnight at 4°C before testing for bone resorbing activity. The cultures were incubated for a further 48 h and the calcium content of the media was measured by automated colorimetric analysis (Gitelman, 1967). In order to ascertain the potency of the tumour cell media recombinant human IL1 beta was tested on the bone resorbing system in parallel.

*Thymocyte proliferation assay*

Interleukin 1-like activity was measured by its capacity to enhance the mitogen-stimulated proliferation of mouse thymocytes (the 'lymphocyte activation factor' (LAF) assay, Gery *et al.*, 1972). Briefly, thymocytes from 7-week old C3H-HeJ mice (Harlan Olac, Blackthorn, UK) were plated in 96 well culture plates (Sterilin) at $1.5 \times 10^6$ per well in $100\,\mu l$ RPMI 1640 containing 5% complement-inactivated FCS, 4 mM 2-mercaptoethanol, and $0.5\,\mu g\,ml^{-1}$ concanavalin A. The cells were cultured for 48 h and $^3$H-thymidine (Amersham International), $0.5\,\mu Ci$ per well, was added for the last 6 h. Incorporation of $^3$H-thymidine into 5% trichloroacetic acid-insoluble material was measured by scintillation counting.

*Chondrocyte collagenase assay*

Rabbit articular chondrocytes were prepared by a method adapted from Evequoz *et al.* (1984). Slices of articular cartilage from the knee and shoulder joints of 2-week old New Zealand white rabbits were sequentially digested with 0.5% hylaluronidase (Sigma) for 20 min at 37°C, followed by 0.25% trypsin and 0.1% bacterial collagenase (Sigma) for 1 h at 37°C in serum free MEM, then 0.1% bacterial collagenase for 2 h in the presence of 10% FCS.

The cells obtained were washed twice and grown to confluence in MEM with 10% FCS. Cell suspensions from the cultures were prepared by trypsin digestion (0.25%), and inoculated into 16 mm wells in 24-well culture plates at 200,000 cells per well. The culture medium was removed when the cells were confluent, and 0.5 ml aliquots test preparations diluted in MEM incubated in triplicate wells for 48 h.

The supernatants were assayed for collagenase activity using thermally reconstituted $^3$H-acetylated rat skin collagen fibrils (Cawston & Barrett, 1979). Latent collagenase in triplicate $50\,\mu l$ samples of the supernatants was activated with $20\,\mu l$ of 10 mM 4-amino phenyl mercuric acetate (Sigma) and incubated with collagen fibrils (10,000 dpm/tube) for 5 h at 37°C. Collagenase activity was calculated from the release of radioactivity into the solution, and expressed as $U\,ml^{-1}$ where 1 U digests $1\,\mu g$ of collagen per minute.

*Tumour necrosis factor assay*

The bioassay for TNF activity is based on cytotoxicity to the murine connective tissue cell line L929 (Flick & Gifford, 1984). Cytotoxicity was measured by the quantitative uptake of methylene blue stain by the cells after fixation (Currie, 1981).

L929 cells were seeded into 96-well microtitre plates (Sterilin) at $4 \times 10^4$ cells per well. After overnight attachment, the medium was removed and replaced with fresh serum-free MEM containing actinomycin D ($1\,\mu g\,ml^{-1}$) and serial dilutions of the standard human recombinant TNF alpha (Genzyme, Suffolk, UK) and tumour cell media at dilution from 1:1 to 1:256 in MEM. After a 24 h incubation the medium was discarded and the cells were fixed with 5% normal formal-saline and stained with methylene blue. The stain was eluted into $100\,\mu l$ 0.1 M HCl, and the absorbance was measured at 650 nm on an automated multi-channel spectrophotometer (Titertek Multiscan, Flow Laboratories, Scotland).

*Stimulation of fibroblast $PGE_2$ synthesis*

Fibroblast cultures were established from normal gingival tissue obtained during routine oral surgery, and grown to confluence in MEM with 10% FCS. The medium was changed twice weekly, until primary cultures were established. The fibroblasts were subcultured weekly by trypsinisation (0.25%), and used between the 4th and 8th passage.

Fibroblasts were seeded in 16 mm culture wells (Sterilin) at 50,000 cells per well. The test preparations were diluted in MEM and 0.5 ml aliquots incubated with the cells for 48 h. $PGE_2$ in the culture media was measured by radioimmunoassay: $^3$H-$PGE_2$ was obtained from Amersham International; $PGE_2$ antiserum from Steranti and $PGE_2$ standards from Sigma.

### Results

*Bone resorption*

The supernatants from all three cell lines caused significant bone resorption, comparable with that produced by MCF. This was partially inhibited by the addition of indomethacin (Figure 1a).

The concentration-dependence of resorbing activity in the tumour cell media is shown in Figure 1b and of recombinant IL1 in Figure 1c.

Addition of anti-IL1 to the tumour cell line media and MCF completely inhibited their osteolytic activity (Figure 1d).

*Thymocyte proliferation*

The tumour cell culture media enhanced the proliferation of Con A-induced mouse thymocytes, indicating the production of IL-1 like activity (Figure 2). The stimulation produced was similar to that obtained by the preparation of MCF.

*Chondrocyte collagenase production*

All three cell lines stimulated collagenase production by rabbit articular chondrocytes in culture (Figure 3). None of the tumour cell-conditioned media had collagenase activity.

*L929 cytotoxicity assay for TNF*

The concentration of TNF that gave 50% maximal cell killing (1 U) was equivalent to $150\,pg\,ml^{-1}$ of recombinant human TNF alpha. There was no detectable TNF activity in any of the tumour cell culture media in any of the concentration tested (results not shown).

*Fibroblast $PGE_2$ synthesis*

All three tumour cell media contained negligible $PGE_2$ ($<0.1\,ng\,ml^{-1}$), but enhanced $PGE_2$ synthesis on addition to fibroblasts. The stimulation was comparable to that obtained with the MCF preparation (Figure 4).

*HPLC*

The HPLC fractions were assayed for IL1 activity in chondrocyte collagenase assay. The results (Figure 5) showed a single major peak of stimulator activity which co-eluted with that in the MCF preparation at an Mr of 15–17,000.

**Figure 1** **(a)** The effects of cell supernatant (diluted 1:10) (with and without indomethacin ($10^{-6}$ M) on bone resorption. Crossed hatched bars show the effect of indomethacin. Mononuclear cell factor (MCF) and $PGE_2$ ($10^{-6}$ M) were used as positive controls. The results are shown as mean ±s.d. ($n=5$ bones per group); * $= P<0.001$ compared with the control group. **(b)** The effects of conditioned media from the cell lines (diluted 1:40, 1:20, 1:10 and 1:5) $PGE_2$ ($10^{-6}$ M) was used as positive control. The results are shown as mean ±s.d. ($n=5$ bones per group); *$P<0.05$ compared with the control group. HN11 -◐-; HN12 -O-; HN15 -●-. **(c)** The effect human recombinant IL1 at the following concentrations; 0.01, 0.1, 1 and 10 nM on bone resorption. $PGE_2$ ($10^{-6}$ M) was used as positive control. The results are shown as mean ±s.d. ($n=5$ bones per group); *$P<0.05$ compared with the control group. **(d)** The effects of tumour cell culture media and MCF (diluted 1:10) on bone resorption and the effect of the same media in the presence of anti-human IL1 on bone resorption (crossed hatched bars) $PGE_2$ ($10^{-6}$ M) was used as positive control. The results are shown as mean ±s.d. ($n=5$ bones per group); * $= P<0.001$ compared with the control group. The bone resorbing activity of MCF and the three cell lines was abolished by the addition of anti-human IL1.

**Figure 2** The effects of cell supernatant (diluted 1:10) on the LAF assay. MCF (diluted 1:10) was used as a positive control. The results are shown as mean ±s.d. ($n=5$). *$P<0.001$.

**Figure 3** The effects of cell supernatant (diluted 1:10) on the production of collagenase by chondrocytes. MCF (diluted 1:10) was used as a positive control. The results are shown as mean ±s.d. ($n=5$). *$P<0.001$.

**Figure 4** The effects of cell supernatant (diluted 1:10) on $PGE_2$ production by fibroblasts. MCF (diluted 1:10) was used as a positive control. The results are shown as mean ±s.d. ($n=5$). *$P<0.001$.

### Discussion

These results have demonstrated that squamous carcinoma cell lines synthesise an osteolytic factor *in vitro* with the characteristics of the cytokine IL1. These, and similar cell lines which have previously been validated in terms of their karyotypes, ultrastructure and growth as xenografts, are known to synthesise osteolytic activity which was not attributable to prostaglandin $E_2$, and was largely unaffected

**Figure 5** The effects of HPLC fractions of the cell supernatants (diluted 1:40) on collagenase production by chondrocytes. HN11 ●——●; JH12 ○——○.

by the inclusion of indomethacin in the cultures (Tsao *et al.*, 1983).

We have shown that the osteolytic factor is non-dialysable (i.e. has a mol. wt in excess of ~10,000) and that the culture medium from the cell lines also contains a non-dialysable factor which has co-mitogenic activity in the thymocyte proliferation (LAF) assay (Figure 2), stimulates collagenase synthesis by articular chondrocytes (Figure 3) and stimulates $PGE_2$ synthesis in fibroblast cultures (Figure 4). The LAF assay and the stimulation of chondrocyte collagenase synthesis are the bioassays normally used to detect IL1. The LAF assay will also detect IL2 (Gery *et al.*, 1972), but the stimulation of chondrocyte collagenase synthesis is independent of IL2 (Evequoz *et al.*, 1984), thus indicating IL1 activity in the cell culture media.

We have also demonstrated that there is no TNF activity in the supernatants. Studies with the polyclonal antibody for IL1 showed that the bone resorbing activity of the supernatant was completely blocked, indicating most of the osteolytic activity in the cell supernatants was IL1-like. Furthermore the stimulation of fibroblast $PGE_2$ synthesis also suggests cytokine activity in the supernatants. This activity in macrophage cultures, first reported by Dayer *et al.* (1977), was later shown to be due to IL1 (Mizel *et al.*, 1981). More evidence for the identity of the osteolytic factor as IL1 was provided by the inhibitory effect of adding anti-human IL1 (Figure 1d).

Fractionation of the culture media by HPLC (Figure 5) showed that the stimulatory activity in the chondrocyte collagenase assay co-eluted with MCF at an Mr of 15–17,000. This is also consistent with its identity as IL1 (Dinarello *et al.*, 1977).

Recently another cytokine, TNF, has also been shown to share several characteristicsof IL1 (reviewed by Le & Vilcek, 1987) including the stimulation of bone resorption *in vitro* (Bertolini *et al.*, 1986). As the mol. wt of TNF (17,000) is similar to that of IL1 it is possible that TNF contributed to the stimulation of bone resorption and $PGE_2$ synthesis by fibroblasts. However, no TNF-like activity was detectable in the tumour cell media over a wide range of dilutions. Furthermore TNF does not exhibit activity in the LAF assay (Dinarello *et al.*, 1985) and does not stimulate metalloproteinase synthesis by chondrocytes (Schnyder *et al.*, 1987). TNF does not, therefore appear to contribute to the osteolytic activity synthesised by the tumour cell *in vitro*.

Another family of peptides, tumour-derived transforming growth factors (TGF), have recently been shown to stimulate bone resorption (Tashjian *et al.*, 1985; Ibbotson *et al.*, 1986) and are thought to be potential mediators of tumour-induced osteolysis. However, TGF does not show IL1-like activity in the LAF and chondrocyte collagenase assays, and is therefore unlikely to contribute to the activity which co-elutes with IL1 on HPLC.

The initial stages of tumour-induced bone destruction are mediated by osteoclastic activity, whereas the later stages can involve direct invasion and destruction of the bone by tumour cells alone (Galasko & Bennett, 1976; Carter, 1985). There is much evidence that prostaglandins are synthesised by human carcinomas (Bennett *et al.*, 1975; Dowsett *et al.*, 1976; Tsao *et al.*, 1981, 1983) and, in view of their potent bone resorbing activity, notably $PGE_2$ (Klein & Raisz, 1970), they have been considered prime candidates as osteolytic mediators. The experiments reported by Tsao *et al.* (1983) showed that although explanted tumour fragments synthesised amounts of $PGE_2$ enough to account for the majority of bone resorption *in vitro*, the carcinoma cell lines synthesised little prostaglandins but significant prostaglandin-independent osteolytic factors. The experiments reported here show that IL1 may account for this PG-independent osteolytic activity.

Although IL1 is an extremely potent resorbing peptide and may contribute to osteolysis by the tumour tissue, its ability to stimulate prostaglandin synthesis in connective tissue cells may be of equal or greater importance. Fibroblasts are target cells for IL1 action and respond by synthesising prostaglandins – notably $PGE_2$ (Dayer *et al.*, 1977; Mizel *et al.*, 1981), $PGF_2$ and prostacyclin (Harvey *et al.*, 1984). In the squamous cell carcinoma, therefore, secretion of IL1 by the carcinoma cells is likely to stimulate prostaglandin synthesis by the infiltrating stromal cells such as fibroblasts and capillary endothelial cells.

We propose, therefore that IL1 secretion by the carcinoma cells can cause bone resorption by two mechanisms: stimulation of osteoclast activity, probably mediated by osteoblasts (Thomson *et al.*, 1986), and indirectly by the stimulation of prostaglandin synthesis in stromal cells infiltrating or surrounding the tumour.

### References

BENNETT, A., McDONALD, A.M., SIMPSON, T.S. & STAMFORD, I. (1975). Breast cancer, prostaglandins and bone metastases, *Lancet*, **i**, 1218.

BERTOLINI, D.R., NEDWIN, G.E., BRINGMAN, T.S., SMITH, D.D. & MUNDAY, G.R. (1986). Stimulation of bone resorption and inhibition of bone formation *in vitro* by human tumour necrosis factors. *Nature*, **319**, 516.

CARTER, R.L. (1985). Patterns and mechanisms of localised bone invasion by tumours: studied with squamous cell carcinoma of the head and neck. *CRC Critical Rev. Clin. Lab. Sci.*, **22**, 275.

CAWSTON, T.E. & BARRETT, A.J. (1979). A rapid and reproducible assay for collagenase using [1-$^{14}$C] acetylated collagen. *Analyt. Biochem.*, **99**, 340.

CURRIE, G.A. (1981). Platelet-derived growth factor requirements of *in vitro* proliferation of normal and mesenchymal cell. *Br. J. Cancer*, **43**, 335.

DAYER, J.M., ROBINSON, D.R. & KRANE, S.M. (1977). Prostaglandin production by rheumatoid synovial cells: Stimulation by a factor from human mononuclear cells. *J. Exp. Med.*, **145**, 1399.

DINARELLO, C.A., RENFER, L. & WOLFF, S.M. (1977). Human leukocytic pyrogen purification and development of a radio-immunoassay. *Proc. Natl Acad. Sci. USA*, **74**, 4624.

DINARELLO. C.A. (1985). An update on human interleukin-1 from molecular biology to clinical relevance. *J. Clin. Immunol*, **5**,287.

DOWSETT, M., EASTY, G.C., POWLES, T.J., EASTY, D.M. & NEVILLE, A.M. (1976). Human breast tumour-induced osteolysis and prostaglandins. *Prostaglandins*, **11**, 447.

EVEQUOZ, V., BETTEN, F., KRISTENSEN, F. & 5 others (1984). Interleukin 2-independent stimulation of rabbit chondrocyte and prostaglandin $E_2$ production by and interleukin 1-like factor. *Eur. J. Path.*, **14**, 490.

FLICK, D.A. & GIFFORD, G.E. (1984). Comparison of *in vitro* cell cytotoxic assay for tumour necrosis factor. *J. Immunol. Methods*, **68**, 167.

GALASKO, C.S.B. & BENNETT, A. (1976). Relationship of bone destruction in skeletal metastases to osteoclast activation and prostaglandins. *Nature*, **263**, 508.

GERY, I., GERSHON, R.K. & WAKSMAN, B.H. (1972). Potentiation of the T-lymphocyte response to mitogen. 1. The responding cells. *J. Exp. Med.*, **136**, 128.

GITELMAN, H.J. (1967). An improved automated procedure for the determination of calcium in biological specimens. *Analyt. Biochem.*, **18**, 521.

GOLDHABER, P. (1960). Enhancement of bone resorption in tissue culture by mouse fibrosarcoma. *Proc. Amer. Ass. Cancer Res.*, **3**, 113 (*abstract*).

GOWEN, M., WOOD, D.D., IHRIE, E.J., McGUIRE, M.K.B. & RUSSEL, G.G. (1983). An interleukin 1-like factor stimulates bone resorption *in vitro*. *Nature*, **306**, 378.

HARVEY, W., FOO, G.-C., GORDON, D., MEGHJI, S., EVANS A. & HARRIS, M. (1984). Evidence for fibroblasts as the major source of prostacyclin and prostaglandin synthesis in man. *Arch. Oral Biol.*, **29**, 223.

HORTON, J.E., RAISZ, L.G., SIMMONS, H.A., OPPENHEIM, J.J. & MERGENHAGEN, S.E. (1972). Bone resorbing activity in supernatant fluid from human cultured peripheral blood leukocytes. *Science*, **177**, 793.

IBBOTSON, K.J., D'SOUZA, S.M., NG, K.W. & 4 others (1983). Tumour-derived growth factor increases bone resorption in tumour associated with humoral hypercalcaemia of malignancy. *Science*, **221**, 1292.

IBBOTSON, K.J., HARROD, J., GOWEN, M. & 5 others (1986). Human recombinant transforming growth factor a stimulates bone resorption and inhibits formation *in vitro*. *Proc. Natl Acad. Sci., USA*. **83**, 2228.

KLEIN, D.C. & RAISZ, L.G. (1970). Prostaglandins: stimulation of bone resorption in tissue culture. *Endocrinology*, **86**, 1436.

LE, J. & VILCEK, J. (1987). Biology of diseases. Tumour necrosis factor and interleukin 1: cytokines with multiple overlapping biological activities. *Lab. Invest.*, **56**, 234.

LUGER, T.A., STADLER, B.M., KATZ, S.I. & OPPENHEIM, J.J. (1981). Epidermal cell (keratinocyte)-derived thymocyte-activating factor (ETAF). *J. Immunol.*, **127**, 1493.

MEGHJI, S., SCUTT, A. & HARVEY, W. (1987). Stimulation of bone resorption by lipoxygenase products *in vitro*. *J. Dent. Res.*, **66**, 238.

MIZEL, S.B., DAYER, J.M., KRANE, S.M. & MERGEHAGEN, S.E. (1981). Stimulation of rheumatoid synovial cell collagenase and prostaglandin production by partially purified lymphocyte activity factor (interleukin 1). *Proc. Natl Acad. Sci. USA*. **78**, 2474.

MUNDY, G.R., RAISZ, L.G., COOPER, R.A., SCHECHTER, G.P. & SALMON, S.E. (1974). Evidence for the secretion of an osteoclast stimulation factor from myeloma. *N. Engl. J. Med.*, **291**, 1041.

PORTEDER, H., MATEJKA, M., ULRICH, W. & SINZINGER, D. (1984). The cyclo-oxygenase and lipoxygenase pathway in human oral cancer tissue. *J. Maxillo. Surg.*, **12**, 145.

SCHNYDER, J., PAYNE, T. & DINARELLO, C.A. (1987). Human monocyte and recombinant interleukin 1's are specific for the secretion of metalloproteinases from chondrocytes. *J. Immunol.*, **138**, 496.

TASHJIAN, A.H.J., VOELKEL, E.T., LEVINE, L. & GOLDHABER, P. (1972). Evidence that bone-resorption-stimulating factor produced by mouse fibrosarcome cells is prostaglandin $E_2$: a new model for the hypercalcaemia of cancer. *J. Exp. Med.*, **136**, 1329.

TASHJIAN, A.H., VOEKEL, E.F., LAZZARO, M. & 5 others (1985). Alpha and beta human transforming growth factors stimulation prostaglandin production and bone resorption in cultured mouse calvaria. *Proc. Natl Acad. Sci., USA*, **82**, 4535.

THOMSON, B.M., SAKLATVALA, J. & CHAMBERS, T.J. (1986). Osteoblasts mediate interleukin 1 stimulation of bone resorption by rat osteoclasts. *J. Exp. Med.*, **164**, 104.

TSAO, S.W., BURMAN, J.F., EASTY, D.M., EASTY, G.C. & CARTER, R.L. (1981). Some mechanisms of local bone destruction by squamous cell carcinoma of head and neck. *Br. J. Cancer*, **43**, 392.

TSAO, M., BURMAN, J.F., PITTMAN, M.R. & CARTER, R.L. (1983). Further mechanisms of bone destruction by squamous cell carcinoma of the head and neck: The role of host stroma. *Br. J. Cancer*, **48**, 697.

VOELKEL, E.F., TASHJIAN, A.H. Jr, FRANKLIN, R, *et al.* (1975). Hypercalcaemia and tumour-prostaglandins. The VX2 carcinoma model in the rabbit. *Metabolism*, **24**, 973.

ZANELLI, J.M., LEA, D.J., & NISBET, J.A. (1969). A bioassay method *in vitro* for parathyroid hormone. *J. Endocrinol.*, **43**, 36.